# Glucocorticoid Receptor-Dependent Gene Regulatory Networks

**DOI:** 10.1371/journal.pgen.0010016

**Published:** 2005-08-05

**Authors:** Phillip Phuc Le, Joshua R Friedman, Jonathan Schug, John E Brestelli, J. Brandon Parker, Irina M Bochkis, Klaus H Kaestner

**Affiliations:** 1 Department of Genetics, University of Pennsylvania School of Medicine, Philadelphia, Pennsylvania, United States of America; 2 Department of Pediatrics, University of Pennsylvania School of Medicine, Philadelphia, Pennsylvania, United States of America; 3 Center for Bioinformatics, University of Pennsylvania School of Medicine, Philadelphia, Pennsylvania, United States of America; North Carolina State University, United States of America

## Abstract

While the molecular mechanisms of glucocorticoid regulation of transcription have been studied in detail, the global networks regulated by the glucocorticoid receptor (GR) remain unknown. To address this question, we performed an orthogonal analysis to identify direct targets of the GR. First, we analyzed the expression profile of mouse livers in the presence or absence of exogenous glucocorticoid, resulting in over 1,300 differentially expressed genes. We then executed genome-wide location analysis on chromatin from the same livers, identifying more than 300 promoters that are bound by the GR. Intersecting the two lists yielded 53 genes whose expression is functionally dependent upon the ligand-bound GR. Further network and sequence analysis of the functional targets enabled us to suggest interactions between the GR and other transcription factors at specific target genes. Together, our results further our understanding of the GR and its targets, and provide the basis for more targeted glucocorticoid therapies.

## Synopsis

Glucocorticoids are essential steroid hormones, and synthetic glucocorticoids are widely prescribed for a variety of medical conditions. Understanding the mechanism by which glucocorticoids act requires knowing the direct target genes whose expression levels are modulated by the glucocorticoid signaling pathway. In this publication, Le and colleagues have utilized two high-throughput techniques to determine genes directly regulated in vivo by the glucocorticoid receptor (GR). RNA and chromatin were extracted from the livers of mice injected with the synthetic glucocorticoid dexamethasone and compared to control littermates. The analysis of RNA expression levels generated a list of genes differentially expressed after addition of dexamethasone. The analysis of the chromatin produced a list of gene promoter sequences where the GR was bound to DNA. By intersecting the two lists, the researchers obtained a list of genes that are directly controlled by the GR, including several previously known targets. This list of direct targets was then used as the basis for complex pathways and sequence analyses, which suggested several interactions between the GR and other transcription factors. This study provides an evaluation of a medically important signaling pathway and serves as a model for future analyses of transcriptional regulation.

## Introduction

Glucocorticoids are essential steroid hormones that are secreted by the adrenal cortex and affect multiple organ systems. Among these effects are the ability to depress the immune system, repress inflammation, and help mobilize glucose in the fasting state. Glucocorticoids and their synthetic analogs are widely prescribed for adrenocortical insufficiency and as an immune suppressant/anti-inflammatory agent, but their systemic effects can often be debilitating. An understanding of the genes regulated by the glucocorticoid signaling pathway may lead to more targeted therapies, thereby preventing unwanted side effects.

Glucocorticoids act via a signaling pathway that involves the glucocorticoid receptor (GR), a member of the nuclear receptor superfamily of ligand-activated transcription factors [1,2]. In the absence of glucocorticoids, the GR is sequestered in the cytoplasm by a protein complex that includes heat shock protein 70 (HSP70) and HSP90. When glucocorticoids are present, they traverse the plasma membrane and bind to the GR, allowing the GR to dissociate from its chaperone proteins and translocate to the nucleus. Within the nucleus, the ligand-bound GR can bind to DNA as a monomer or as a dimer to palindromic glucocorticoid response elements (GREs) and modulate transcription [3–7].

The mechanisms of action of the ligand-bound GR are fairly complex, including the ability to both activate and repress transcription, and to interact with other transcriptional regulators such as activating protein-1 (AP-1) and nuclear factor kappa B (NF-κB) (reviewed in McKay and Cidlowski [5]). The net effect of glucocorticoid administration on a particular target gene is likely dependent upon the other transcription factors present on the target gene's promoter or enhancer(s). Specifically, the integration of multiple signaling pathways can occur at glucocorticoid response units (GRUs), which consist of a combination of a GRE and other transcription factor binding sites. Examples of these include GRUs in the promoters of the *phosphoenolpyruvate carboxykinase* *(Pck)* and *carbamoylphosphate synthetase* *(Cps)* genes [8–10]. Thus, understanding the complete nature of glucocorticoid action requires knowing not only the set of genes bound and regulated by the GR, but also the transcription factors that may interact with the GR, and the loci where these interactions occur.

To better understand glucocorticoid signaling, RNA expression profiling after glucocorticoid administration has been performed by several groups [11–16]. However, it is impossible to establish which differentially expressed genes are direct targets of the GR and which are controlled by downstream effectors. To address this limitation, Wang and colleagues have developed a technique termed “ChIP scanning,” which involves screening the promoter region of each putative target gene individually using chromatin immunoprecipitation (ChIP) and quantitative real-time PCR (QPCR) [17]. Unfortunately, this technique is not high-throughput, but instead involves designing multiple primer sets for each potential target gene individually. Thus, this technique is not suitable for global network analysis.

A modification of microarray technology utilizes spotted promoter regions instead of cDNA sequences. After ChIP with an antiserum raised against a particular transcription factor, the immunoprecipitated DNA is amplified, labeled, and hybridized against these promoter microarrays. Spots that are brighter in the immunoprecipitated channel than the control represent promoter sequences to which the transcription factor is bound. This technique, termed “genome-wide location analysis,” or “ChIP-on-Chip” has been utilized to determine binding sites for several transcription factors in yeast [18,19], and higher organisms [20–25]. However, binding data alone do not prove that the transcription factor of interest is important in the regulation of a particular target gene. This is especially true in higher eukaryotes, where many genes are regulated by dozens of partly redundant transcription factors [26]. Therefore, in order to identify direct targets of a given transcription factor that are dependent on the regulatory protein in question, a combination of location analysis and expression profiling is required.

Given the importance of the glucocorticoid signaling pathway in biology and medicine, we have undertaken a study to determine functional GR targets. We performed parallel mRNA expression profiling and location analysis on livers of fasted mice injected with glucocorticoids and compared the results with those of fed controls. Individually, the expression analysis and the location analysis each produced lists of genes that included many known or suspected GR targets. By combining the expression and binding data, we were able to identify 53 direct functional GR targets, many of which are novel. In addition, network and sequence analysis of the GR targets independently suggested functional interactions between the GR and several other transcription factors. Through these experiments we have extended the understanding of the complexity of the genetic networks modulated by glucocorticoids.

## Results

### Identification of GR Targets by Expression Profiling

**Figure 1:**
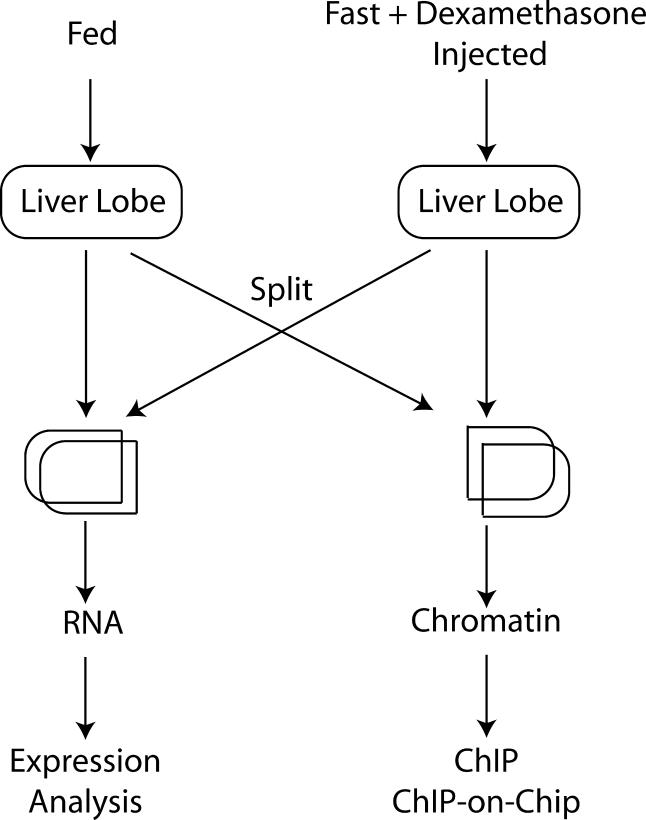
Experimental Paradigm for Orthogonal Analysis of Glucocorticoid Receptor Targets Treatment mice were fasted overnight, then given intraperitoneal injections with dexamethasone. Treatment and control liver lobes were split in half and processed for both RNA and chromatin. RNA was subjected to microarray analysis using the PancChip 5.0 cDNA microarray, which contains over 13,000 transcripts. Location analysis was performed by immunoprecipitating with antiserum raised against the GR. ChIP material was amplified, fluorescently labeled, and hybridized against sheared genomic DNA using the Mouse PromoterChip BCBC-3.0 promoter microarray, which contains approximately 7,000 genomic promoter elements.

The experimental paradigm we chose to use for parallel expression profiling and location analysis is outlined in Figure 1. Treated mice were fasted overnight and injected with dexamethasone, a synthetic glucocorticoid. Control mice were fed ad libitum and not injected with vehicle, as this would generate a stress response. Treated and control mice were sacrificed, and the left lobe of their livers was removed for parallel expression and genome-wide location analysis.

Several alternative experimental designs were initially considered and ultimately discarded. First, we opted to evaluate the networks controlled by glucocorticoids in hepatocytes in mice and not in hepatoma cell lines grown in culture. While this approach was technically more challenging, it was necessary because available cell lines do not reflect the metabolic regulation of hepatic gene expression accurately [27,28]. Second, it was important to compare mice in different feeding states due to the significant differences in the levels of insulin, glucagon, and glucocorticoids that are present in each state. One alternative design would have been to compare dexamethasone-injected fed mice to fed controls. When we performed a preliminary expression analysis using this design, the levels of many well-known GR targets, such as *Pck* and *insulin-like growth factor binding protein 1* *(Igfbp1),* were unchanged (unpublished data). These results are consistent with the well-described ability of insulin signaling to inhibit glucocorticoid activation of many targets, such as *Pck, Igfbp1,* *glucose-6-phosphatase (G6pc),* and *6-phospho-2-fructokinase (Pfk2)* (reviewed in [29]). In addition to the presence of the inhibitory effects of insulin, an experimental design in which both groups are fed risks a significant loss of sensitivity due to a lack of fasting-induced signals that act synergistically with glucocorticoids, such as glucagon. Another alternative design would have been to compare mice fasted and injected with dexamethasone to fasted control mice. However, since endogenous glucocorticoids are released in the fasted state, the chromatin from the control samples could not serve as negative controls for the location analysis. Therefore, we elected to compare dexamethasone-injected fasted mice to fed controls.

Treated and control liver lobes were split in half. RNA was extracted from one half, while chromatin was prepared from the remaining half. Reverse-transcription QPCR (RT-QPCR) was performed to measure relative expression levels of several targets known to be induced by glucocorticoids in fasted mice. As expected, the mRNA levels of these targets, including *Pck, tyrosine aminotransferase (Tat),* and *Igfbp1,* were induced between 6- and 60-fold relative to the levels in the fed control mice (unpublished data).

The RNA was then used to perform a microarray hybridization using the PancChip 5.0 expression microarray [30]. Of the 13,000 transcripts on the array, approximately 1,300 unique genes were differentially expressed between the two conditions at a false discovery rate of 10% (complete dataset available in Datasets S1 and S2). Of those, about 30% were up-regulated in the dexamethasone-injected livers, while the majority was repressed. Again, the list of differentially expressed genes included many known GR targets, including *Pck, Igfbp1,* and *metallothionein 2 (Mt2)* (unpublished data). However, it is unclear which of these differentially expressed genes are direct targets of the GR.

### Global Analysis of GR Occupancy In Vivo

**Figure 2:**
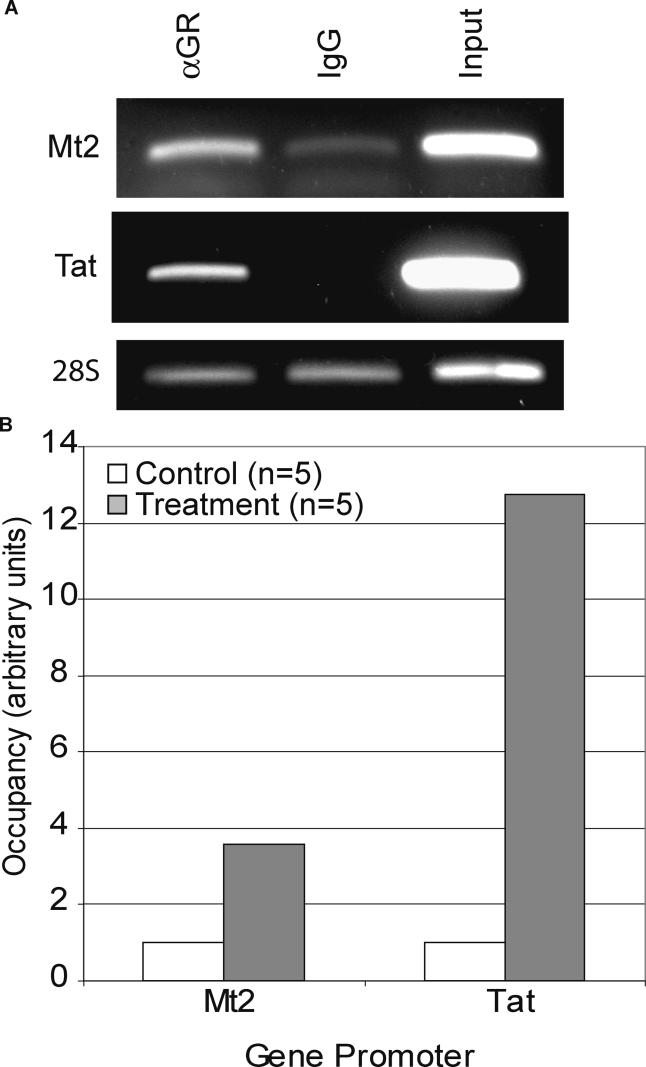
ChIP Identifies Known GR Targets in Liver (A) Agarose gel electrophoresis of PCR products for the known GREs in *Mt2* and *Tat* confirm the specificity of the anti-GR antibody (sc-1002, Santa Cruz) compared to the control preimmune IgG. The PCR product for the genomic locus encoding the 28S ribosomal RNA shows that equal amounts of DNA were loaded into each reaction. (B) QPCR was used to measure the enrichment of *Mt2* and *Tat* in chromatin immunoprecipitated with the anti-GR antiserum from five dexamethasone-treated samples compared to five fed controls, as described in Materials and Methods. The 28S PCR product was used to normalize the samples.

Chromatin immunoprecipitation, performed using an antiserum raised against the GR, was compared to preimmune rabbit IgG to assess the specificity of the antibody at two known GR targets, *Mt2* and *Tat* (Figure 2A). This antibody was then utilized in immunoprecipitations with chromatin from both the dexamethasone-treated and the fed control samples. GR occupancy on those same two targets, as measured by QPCR, was increased by approximately 4-fold and 13-fold, respectively, confirming the efficiency of the ChIP (Figure 2B). Next, we amplified the immunoprecipitated DNA by two rounds of ligation-mediated PCR, and confirmed that the enrichment of the known GR targets was maintained after amplification (unpublished data). Amplified samples were then fluorescently labeled and hybridized against sheared genomic DNA on the Mouse PromoterChip BCBC-3.0 promoter microarray, which contains PCR amplicons of two promoter regions for over 3,300 genes important in liver function. The first PCR amplicon, called tile 1, is approximately 1 kilobase (kb) in length and is located immediately upstream of the putative transcriptional start site. The second amplicon, tile 2, is approximately 2 kb in length and is located immediately upstream of tile 1. In total, we spotted onto the microarray approximately 3 kb of genomic promoter sequence for each of the 3,300 genes.

**Figure 3:**
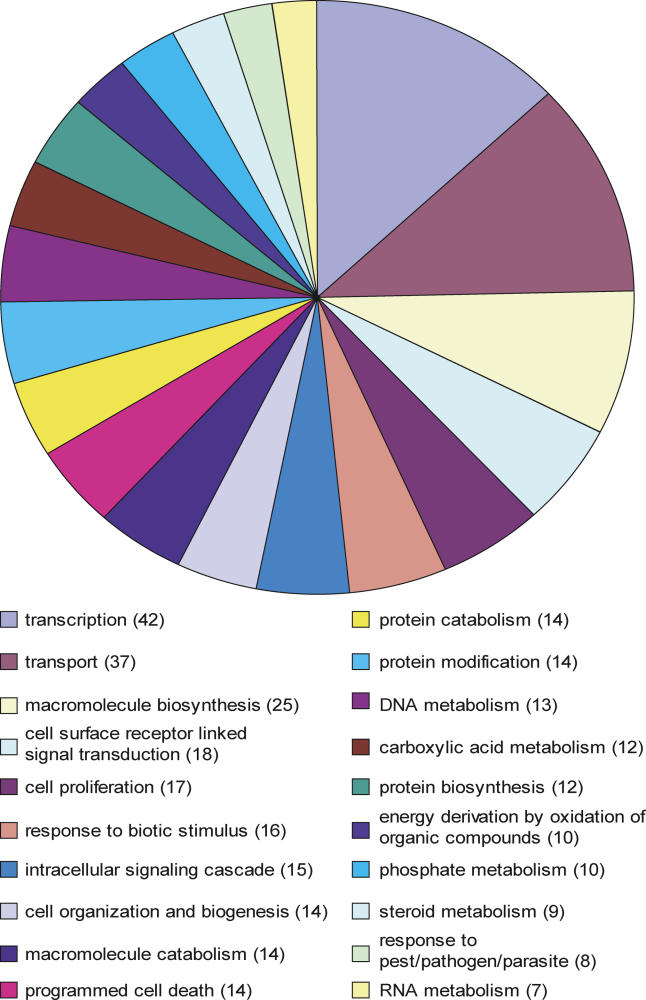
Functional Categories of the Genes Generated by Location Analysis Location analysis was performed using antiserum against the GR. Three treated and five control samples were amplified, fluorescently labeled, and hybridized to the Mouse PromoterChip BCBC-3.0 promoter microarray in a common reference design. Standard statistical methods identified 302 promoter regions significantly enriched in the treated samples compared to the fed controls (see Materials and Methods). The GO level 4 functional category for each gene was retrieved and the top 20 categories are shown. Note that some genes belong to multiple GO categories.

The location analysis identified 318 promoter regions, representing 302 distinct genes, enriched in the immunoprecipitations from dexamethasone-treated samples. This list of GR targets (Table S1) contains many genes previously shown to be regulated by GR, including *Pck*, *Igfbp1, tumor necrosis factor (Tnf),* and hormone-sensitive lipase. The Gene Ontology (GO) Biological Function categories of the GR-bound genes are shown in Figure 3. The GR targets were also assessed for statistical enrichment of GO Biological Function categories. The ten most enriched GO Biological Function categories are shown in Table 1. These categories are dominated by genes important in metabolism, consistent with the function of glucocorticoids in the liver. It is also important to note that within the GO function hierarchy, some genes belong to multiple categories.

**Table 1:**
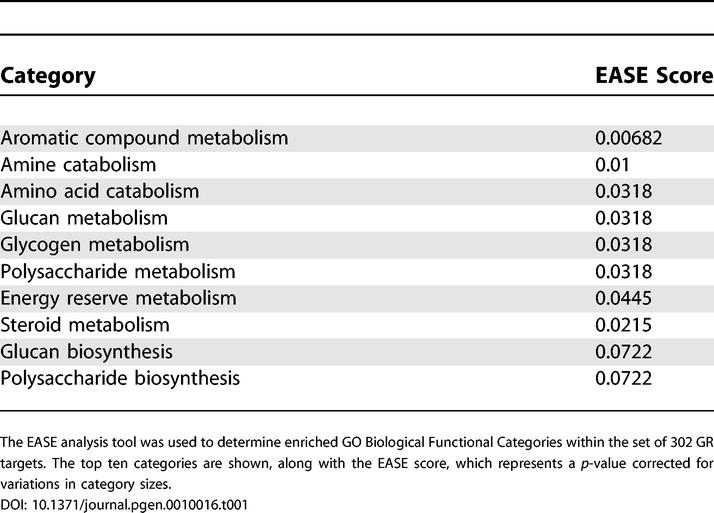
Enriched GO Biological Functional Categories

The EASE analysis tool was used to determine enriched GO Biological Functional Categories within the set of 302 GR targets. The top ten categories are shown, along with the EASE score, which represents a *p*-value corrected for variations in category sizes.

**Figure 4:**
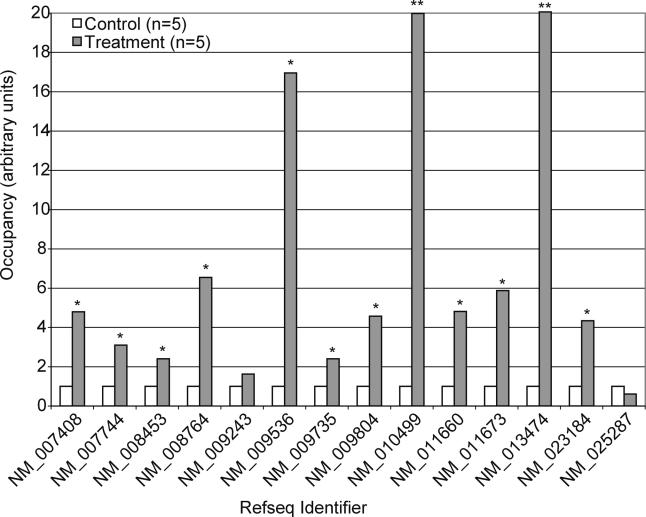
Quantitative PCR Confirms GR Targets Identified by Location Analysis The enrichment of GR targets identified by location analysis was measured in the original immunoprecipitated material by QPCR. The graph shows the fold-enrichment of five dexamethasone-treated samples compared to five fed controls. Of fourteen randomly selected GR targets, 12 showed statistically significant enrichment. Fold-enrichment and *p*-values were calculated as described in Materials and Methods. *p < 0.05, **p < 0.01.

We confirmed several of the targets identified in our location analysis by measuring their enrichment in the original immunoprecipitated DNA using QPCR. Two computational programs, NUBIScan [31] and TESS (http://www.cbil.upenn.edu/tess) were used to identify likely GR binding sites within the spotted promoter regions. QPCR was used to calculate the fold-enrichment of these genomic loci in the unamplified, immunoprecipitated DNA of treated samples compared to controls. The results are shown in Figure 4. Of the 14 samples, 12 (85%) randomly chosen promoters were enriched to the level of significance, confirming the validity of our location analysis. Furthermore, it is possible that the remaining promoters have true GR binding sites, but at loci that do not match the consensus sequence well and thus were not assessed. We also noted that the fold-enrichment measured by QPCR was generally greater than that measured by the promoter microarray. This “compression effect” has been previously described in cDNA and oligonucleotide microarrays [32].

To further evaluate our list of GR-bound promoters, we obtained a list of more than 50 previously published GR targets [5], 12 of which contain one or more GRE consensus sites within the sequences that are present on our promoter array. If the location analysis had produced a random set of 302 genes (302/3300 [approximately 9%]), then of these 12 known targets, approximately one gene could be expected to be on our list. However, of these 12, eight (67%) were identified by our location analysis as occupied by the GR in the liver in vivo (*p* < 2 × 10^−6^), confirming the usefulness of our approach.

### Integrating Expression and Binding Data Results in Functional GR Targets

**Figure 5:**
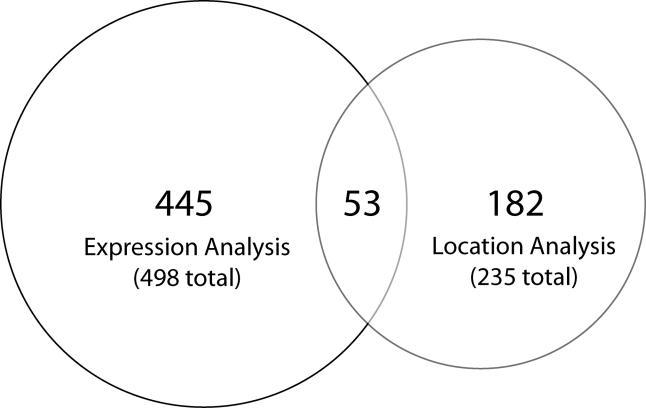
Intersection of Expression Data and Location Analysis Of the genes common to both the cDNA microarray and the promoter microarray, 498 were differentially expressed and 235 were bound by the GR. Intersecting the two lists produced 53 genes in common. This list represents direct, functional targets of the GR in hepatocytes.

In order to determine the subset of genes that are direct and meaningful targets (i.e., are changed in their expression level) of the activated GR in the liver, we identified the overlap of the GR-bound and GR-regulated genes (Figure 5). There are approximately 2,500 genes common to both array platforms used. Of these, 498 were differentially expressed and 235 were bound by the GR. Intersecting the two sets resulted in 53 genes that were both differentially expressed and bound by the GR. These represent direct, functional targets of the activated GR, which we hereafter refer to as the differentially expressed, GR bound (DEB) set. All of these genes are listed in Table 2, and include several published GR targets, including *alcohol dehydrogenase 1 (Adh1),* *Pck,* and *Igfbp1,* as well as likely GR target genes such as *catalase (Cat)* [33]. A more thorough search of the literature and use of expression data from a previously published experiment in a different tissue [34], indicates that 22 of the 53 genes have been shown to be differentially expressed after the addition of glucocorticoid. Thus, 31 of our target genes appear to be novel GR targets.

**Table 2:**
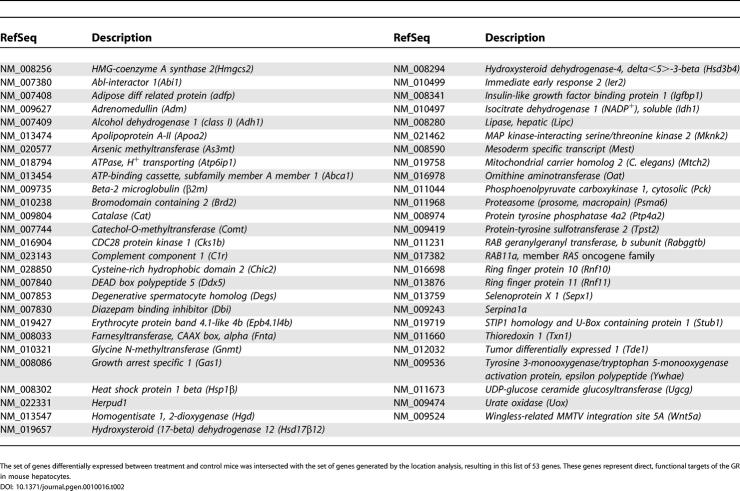
DEB Set

The set of genes differentially expressed between treatment and control mice was intersected with the set of genes generated by the location analysis, resulting in this list of 53 genes. These genes represent direct, functional targets of the GR in mouse hepatocytes.

### Network and Sequence Analysis Suggest Several Potential GR-Protein Interactions and Sites

**Figure 6:**
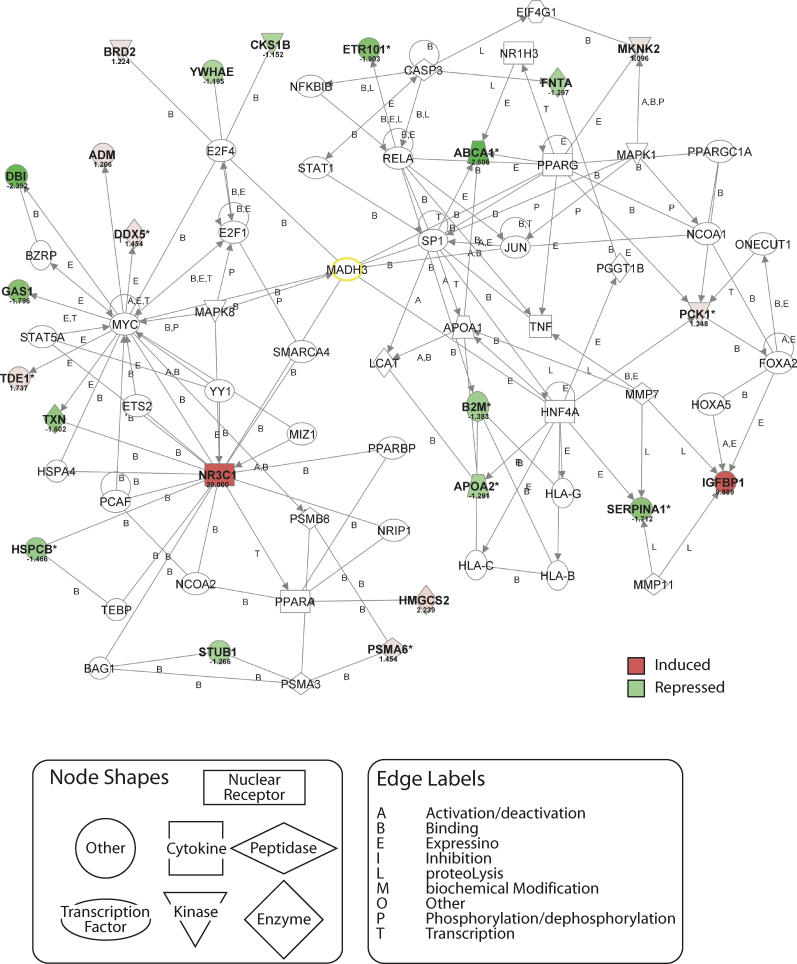
A Regulatory Network for the GR Pathway analysis was seeded with the 53 differentially expressed and GR-bound genes, plus the GR itself, as described in Materials and Methods. Genes in colored, bold text were in the seed set, while all others were brought into the network by the pathway analysis program based on their known relationships to the genes in the seed set. Color indicates induction (red) or repression (green) of expression.

Pathway analysis can be a useful tool to help uncover relationships among genes [35], such as the finding that multiple members of the list may be regulated by the same transcription factor. When seeded with the genes in the DEB set plus the GR itself, pathway analysis produced two networks of genes with functional relationships. These were merged together and are shown in Figure 6. Our data suggest a direct interaction between the GR (NR3C1, large red box; Figure 6) with the DEB gene set (genes in boldface).

Closer inspection revealed that many of the genes added to the network encode transcription factors that are known to physically interact with the GR on the promoters of particular genes. Two well-studied examples include RelA and Jun, which are members of the NF-κB and AP-1 transcriptional complexes, respectively. It has been suggested that the activated GR modulates inflammation and the immune response by physically interacting with NF-κB and AP-1, thereby repressing the activation of many of their targets [5,7]. In fact, recent work has shown that the LIM protein TRIP6 is required for this interaction [36]. Other transcription factors identified by the pathways analysis that are known to interact with the GR to modulate particular genes include *Stat5* [37,38], *FoxA* family members [39,40], *HNF6* [41], *PPARγ* [42], and *Ets* family members [43,44]. This suggests that the other transcription factors in the network, such as Myc and HNF4α, may also interact with the GR to co-regulate target genes. Thus, by combining a database of genetic relationships with a set of direct GR targets, we are able to suggest interactions between the GR and other transcription factors.

Next, we analyzed the sequences of the promoters in the DEB set, searching for enriched transcription factor binding sites. First, we scored the set of vertebrate transcription factor position-specific weight matrices (PWMs) in the TRANSFAC database [45] and in JASPAR [46] against the entire set of tiles on the promoter array. We then determined the ability of individual PWMs (representing single transcription factor binding sites) to distinguish between the DEB set and the background set. The GR PWM was enriched in the DEB set compared to the entire set of promoters on the microarray (*p* < 0.05), as well as compared to the promoters of the 445 genes differentially expressed but not bound by the GR (*p* < 0.01). For instance, at the particular score threshold, 66% of the DEB set contained a sequence surpassing the threshold, compared to only 48% of the 445 genes differentially expressed but not bound by GR.

Interestingly, the GR weight matrices (full-site and half-site) were not the most significantly enriched matrices. Among the remaining matrices representing vertebrate transcription factors, the scores of the matrices for HNF4α and the GATA family of transcription factors were more significant than the GR matrices. It is likely that the significance of the half-site GR consensus sequence, AGAACA [47], is hampered by the high frequency with which it occurs in the background set of DNA, while the full-site matrix scores probably suffer from the fact that true GREs can vary significantly from the published GRE consensus. For example, the functional GREs in the promoter region of *Pck* scored poorly with the consensus full-site matrix. As for the enrichment of other matrices, it may be that in our DEB set the presence of HNF4α binding sites is providing tissue specificity to the glucocorticoid response.

Because it is known that the GR can interact with other transcription factors to modulate gene expression, we searched for significant combinations of binding sites for either GR monomers or dimers and other nearby transcription factors. This analysis resulted in several combinations that were significantly enriched in the DEB set compared to the set of all mouse promoter sequences on the array (Table 3). Once again, the interaction between the GR and the AP-1 transcriptional complex presented itself. Specifically, the combination of a GR monomer (not the dimer) and an AP-1 site occurring within 34 base pairs (bp) was highly enriched in the DEB set (*p* < 10^−7^). This is consistent with published reports that the GR monomer, and not the dimer, physically interacts with AP-1 to repress certain AP-1 target genes [48]. Several other combinations discovered using this analysis have previously been shown to occur, such as the interaction between GR and CCAAT/enhancer binding protein beta (C/EBPβ) [39], GR and YY1 [38] , and GR and Oct-1 [49].

**Table 3:**
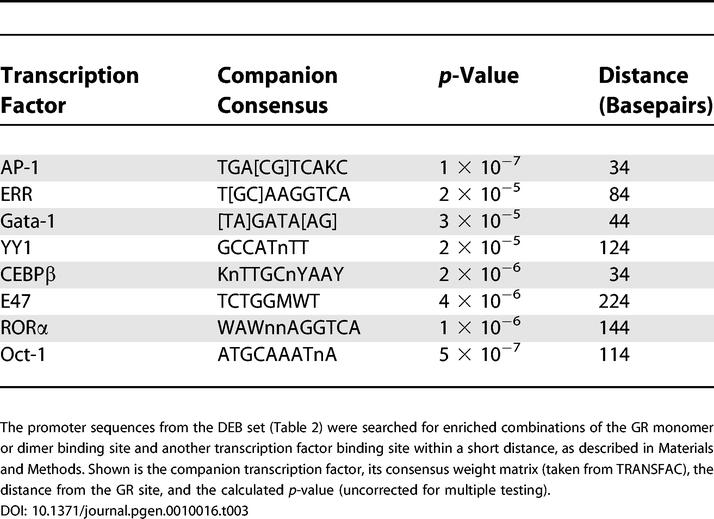
Significant Combinations of GR Monomer/Dimer and Other Transcription Factors

The promoter sequences from the DEB set (Table 2) were searched for enriched combinations of the GR monomer or dimer binding site and another transcription factor binding site within a short distance, as described in Materials and Methods. Shown is the companion transcription factor, its consensus weight matrix (taken from TRANSFAC), the distance from the GR site, and the calculated *p*-value (uncorrected for multiple testing).

We were particularly interested in the potential interaction between the GR and the C/EBP family of transcription factors, since we had previously performed location analysis for C/EBPβ in mouse liver [21]. Of the promoters in the DEB set that contained a high-scoring combination of GR and nearby C/EBP binding site, 11 had been analyzed previously for C/EBPβ binding. Strikingly, five (45%) of these genes, namely *beta-2 microglobulin (β2m), protein-tyrosine sulfotransferase 2 (Tpst2)*, *HSP 1 beta (Hspcβ), ATP-binding cassette sub-family A member 1 (Abca1),* and *hydroxysteroid (17-beta) dehydrogenase 12 (Hsd17β12),* had shown enrichment in that analysis of C/EBPβ ChIP compared to null controls. In other words, of the 11 promoters that were bound and regulated by the GR, had a computationally predicted C/EBP site near a GR site, and were evaluated in an independent experiment, almost half are true C/EBPβ targets. The remaining promoters might be bound by one of the other C/EBP family members expressed in hepatocytes. In any case, this example validates our approach to computationally identifying transcription factors binding to complex GRUs in vivo.

## Discussion

Glucocorticoids are widely used in medical therapy for immunosuppression and as potent anti-inflammatory agents. However, their broad effects on different organ systems often result in debilitating side effects such as bone loss and glucose dysregulation. Knowing the direct, functional targets of the GR increases our understanding of the different mechanisms by which glucocorticoids act and aids the development of directed therapies toward different “arms” of the glucocorticoid response. To approach that aim, we have utilized two high-throughput techniques to obtain orthogonal data sets, allowing us to determine the set of genes regulated by the GR in the liver in vivo. We have found hundreds of genes whose promoters are occupied by the ligand-bound GR and, of those, dozens that are differentially expressed in hepatocytes in the presence of exogenous glucocorticoid. These functional GR targets span the known range of glucocorticoid action, containing both induced and repressed genes, as well as genes involved in metabolism, signal transduction, and the immune response/inflammation. By applying pathway and sequence analysis, we have also generated a list of transcription factors that may interact with the GR to modulate transcription of target genes.

Our expression analysis resulted in approximately 1,300 differentially expressed genes. Of these genes, many are differentially expressed solely due to the change in the feeding state of the animal. However, the alternative experimental designs were unacceptable. In our preliminary comparison of RNA from livers of mice that were fed versus fed and dexamethasone-injected, the lack of differential expression of known GR targets such as *Pck* and *Igfbp1* suggested that an orthogonal analysis with this data set would miss many potential targets. Since the goal of the study was not to produce a list of genes differentially expressed after glucocorticoid administration, which has been previously published by others, but rather to perform an orthogonal analysis utilizing both expression and location data from the same tissue, we deemed it acceptable to trade-off specificity for sensitivity. Moreover, by intersecting the expression and location analysis data, it is likely that false positives within the expression data were removed.

The list of 302 GR-bound promoters contains many genes that are known or suspected GR targets. One important category of target genes are transcriptional regulators, including the homeobox genes *Hoxa13, Hoxb4, Hoxc5, Hoxc9,* and *Hesx1;* *transcription factor 15 (Tcf15), transcription factor 21 (Tcf21),* and *transcription factor AP-4 (Tfap4); Krüppel-like factor 3 (Klf3)* and *Klf15;* as well as *Trp53, Creb1,* and *Fos*. In addition, many metabolic enzymes are present, consistent with the known role of glucocorticoids in regulating glucose metabolism.

One well-known effect of glucocorticoid administration is decreased bone density [50]. Bone density is controlled by a delicate balance between osteoclasts, which resorb bone, and osteoblasts, which mineralize bone. Our location analysis identified many GR targets that modulate bone remodeling, including TNFα and TGFβ signaling pathway members, *adrenomedullin (Adm)*, *calumenin (Calu),* and *annexin 2 (Anxa2)* [51–56]. Of particular interest is the TNF receptor superfamily member osteoprotegerin (OPG), encoded by *Tnfrsf11b*. OPG is a secreted glycoprotein that acts as a decoy receptor for receptor activator of NF-κB ligand, impairing its interaction with receptor activator of NF-κB and thereby inhibiting osteoclast differentiation [57]. Thus, increased levels of OPG promote increased bone density via decreased osteoclast differentiation, while mice that are null for OPG have decreased total bone density, severe bone porosity, and high incidence of fractures [58]. In humans, it has been shown that a deletion of the OPG gene can cause juvenile Paget's disease, an autosomal recessive osteopathy characterized by debilitating fractures and deformities resulting from increased bone turnover [59]. While it has previously been shown that OPG is expressed in liver [60] and that glucocorticoid administration lowers OPG mRNA levels [61], our location analysis provides the first evidence that glucocorticoids repress OPG expression by directly binding to the OPG promoter. Interestingly, the protein encoded by *core binding factor beta (cbfβ),* another GR target identified by our analysis, also plays an important role in bone development, possibly via its interaction with core binding factor alpha, a known regulator of OPG [62,63]. Overall, we have demonstrated that our location analysis identified many known and suspected GR targets that directly affect bone remodeling, one of the major side effects associated with prolonged glucocorticoid administration.

While our microarray-based location analysis was highly accurate, with 85% of the targets confirmed by RT-QPCR, it appears somewhat surprising that only about a quarter of the GR-bound genes (53 of 235 for which we have expression data) were differentially expressed. A small fraction of these targets may be enriched due to nonspecific binding of the ligand-bound GR to DNA, or false positives introduced by the ligation-mediated PCR method used to amplify the material prior to hybridization. Another possibility is that the expression level of the gene may not have been high enough to detect in our expression analysis, and that the use of a more sensitive assay, such as RT-QPCR, would show more GR-bound genes to be differentially expressed. We believe, however, that the majority of these GR targets are likely to be functional, but in a different context, such as another tissue. The specific combination of other transcriptional regulators present, as well as higher-order chromatin structure, likely play a role in determining which glucocorticoid target genes are expressed and regulated. The complex regulatory mechanism present in the GRU of the *Pck* gene is a well-studied example [8]. Insulin signaling represses *Pck* transcription, and this effect is dominant over the transcriptional activation that is promoted by both glucocorticoid and glucagon signaling pathways. Furthermore, *Pck* expression is activated by glucocorticoids in the liver, but repressed by glucocorticoids in adipose tissue [64]. Similar mechanisms may be at work on many of the GR-occupied targets that did not show expression level changes in our particular experiment. It may be possible to expand our list of functional GR targets by incorporating other expression data.

The use of weight matrices to predict transcription factor binding sites without any prior knowledge is highly nonspecific, due to the degenerate nature of the binding sites and the complexity of the eukaryotic genome [65]. Some attempts to refine these predictions have utilized evolutionary conservation [66], while others have used functional information such as coexpression [67]. Our analysis results demonstrate that performing complex sequence analysis on a functional set of promoters can result in more specific predictions regarding transcription factor binding sites and can even allow the prediction of binding sites for other transcription factors that cooperatively regulate target genes.

An alternative to location analysis using a promoter microarray is a technique developed by Impey and colleagues called serial analysis of chromatin occupancy (SACO) [68]. It involves ChIP followed by long serial analysis of gene expression. The difference between the two techniques is significant. Promoter microarrays can only detect ChIP enrichment in the region immediately within and around the sequences spotted on the array. In contrast, SACO is unbiased in the binding sites that it can detect, thus allowing investigators to interrogate the whole genome, including introns and intergenic sequences. However, while the development of the promoter microarray has a large initial expense, all future experiments are relatively inexpensive. This contrasts with SACO, in which the sequencing cost must repeatedly be borne for each additional experiment. Thus, to perform SACO on groups of treated and untreated animals, as we have done here, is prohibitively expensive and time-consuming.

Although this work has expanded the set of genes known to be regulated by the GR, the list remains incomplete. Since glucocorticoids exert different effects on the various organ systems of the body, it is likely that the functional targets of the GR are different in each tissue. This would suggest that repeating this experiment on other glucocorticoid-responsive organs would yield new targets. Alternatively, the target list might remain the same, but the intersection between differentially expressed genes and GR-bound promoters might be different. In the future, it would be extremely useful to determine the sets of genes regulated by individual transcription factors versus those regulated by combinations of factors. It may be that those genes controlled by a more complex regulatory network are key genes in the processes in which they participate. This determination could be achieved by performing location analysis with antisera against different transcription factors and then intersecting the lists generated by each analysis, or by performing a single location analysis after sequential immunoprecipitations with different antisera [69].

In summary, we have performed parallel expression analysis and location analysis on the livers of mice treated with dexamethasone in order to determine direct, functional targets of the GR. Our analysis identified many genes previously shown to be GR targets, many genes that were suspected GR targets, and some novel GR target genes. Some of these genes may be critical for particular aspects of the glucocorticoid response, and would therefore make attractive candidates for targeted therapies. Other target genes may be control points where the integration of multiple signaling pathways occurs via GRUs, such as on the *Pck* promoter. We believe that these targets provide many opportunities for future research, and that this work has moved us one step closer to understanding the complete genetic network modulated by the GR and the set of transcriptional regulators with which it interacts. In addition, this work establishes a paradigm for similar orthogonal analyses, and demonstrates that pathway and sequence analyses can be used to suggest functional interactions between transcription factors on the promoters of particular genes.

## Materials and Methods

The complete expression and location analysis data sets are available as Datasets S1 and S2, respectively. The microarray feature location annotation for the two platforms (Mouse PancChip5.0 and Mouse PromoterChip BCBC-3.0) are downloadable from the EpconDB website (http://www.cbil.upenn.edu/EPConDB/Downloads.shtml).

### Mouse treatment.

Ten adult male CD1 mice were randomly split into two groups. The control mice were fed ad libitum, while the treatment group was fasted overnight and then given intraperitoneal injections of 0.1 mg/g body weight dexamethasone (Sigma-Aldrich, St. Louis, Missouri, United States) diluted in PBS 3 h prior to sacrifice. Control mice were not injected with vehicle (PBS), as this would have caused the release of endogenous glucocorticoid. Control and treatment animals were housed similarly with standard day/night cycles.

### Expression analysis.

RNA was extracted from one half of each liver using TRIZOL (Invitrogen, Carlsbad, California, United States) and lithium chloride precipitation and then analyzed on an Agilent Bioanalyzer 2100 for quality and quantity. All samples showed intact ribosomal bands with a minimum 28S to 18S ratio of 2.0.

For RT-QPCR, 3 μg of total RNA was reverse transcribed using Superscript II (Invitrogen) primed with an oligo-dT primer and then diluted to 300 μL. Each reaction contained 1 μL of diluted cDNA. RT-QPCR was performed in triplicate using a Stratagene MX4000 QPCR machine and Stratagene Brilliant SYBR mix, as per the manufacturer's instructions (Strategene, La Jolla, California, United States). Cycling conditions were 95 °C for 10 min, then 40–45 cycles of 30 s at 95 °C, 1 min at 60 °C, and 30 s at 72 °C, followed by a dissociation curve analysis. Fold enrichments and *p*-values were calculated as using the Relative Expression Software Tool (REST) [70] with 2,000 randomizations and using the expression of TATA box binding protein *(Tbp)* as the normalizing gene. Primer sequences are provided in Table S2.

Microarray hybridizations were performed as previously described [30]. A common reference design was utilized, where the common reference was a mixture of all ten samples. This resulted in ten microarray hybridizations. For each sample, 20 μg of total RNA was reverse transcribed using Superscript III (Invitrogen), oligo-dT primers, and amino-allyl dUTP. The RNA was then degraded and the cDNA coupled to fluorescent Cy3 or Cy5. The samples were hybridized to the PancChip 5.0 cDNA microarray, which contains over 13,000 transcripts (http://www.epcondb.org). Slides were scanned on an Agilent (Palo Alto, California, United States) scanner and analyzed with GenePix 5.0 software. Data were normalized using the SMA add-on [71] in the “R” software package [72] and differentially expressed genes were identified using the SAM software package at 10% false discovery rate [73].

### Chromatin immunoprecipitation.

Chromatin immunoprecipitations were performed as previously described [21]. Half of each mouse liver sample was minced in cold PBS, pushed through a 21-gauge needle, and then crosslinked using 1% formaldehyde for 10 min. The crosslinking was quenched by adding glycine to a final concentration of 0.125 M. The samples were then washed in PBS and Dounce homogenized in ChIP cell lysis buffer (5 mM Pipes [pH 8.0], 85 mM KCl, 0.5% Nonidet P-40, 10 μM leupeptin, and 1 mM PMSF). Nuclei were sedimented and separated from the cellular debris, then placed into nuclear lysis buffer (50 mM Tris HCl [pH 8.1], 10 mM EDTA, 1% SDS, 10 μM aprotinin, 10 μM leupeptin, and 1 mM PMSF). After 10 min on ice, the lysate was sonicated on ice (Sonic Dismembrator Model 100; Fisher, Pittsburgh, Pennsylvania, United States) using three pulses for 20 s at 4–6 W. Insoluble debris was removed by centrifugation, and the supernatant was collected and flash frozen in liquid nitrogen. An input fraction was generated by taking a portion of nonimmunoprecipitated chromatin material and reversing the crosslinking. When visualized on an agarose gel, the DNA produced a smear of approximately 200–600 bp in length.

For each immunoprecipitation, 2 μg of chromatin was used. The chromatin was precleared by incubating with protein-G agarose at 4 °C for 1 h. Antiserum raised against the GR (sc-1002 Santa Cruz, utilized in ChIP assay by [74]) or pre-immune IgG were added (2 μg each) and the samples rotated at 4 °C overnight. Immunoprecipitates were isolated by incubating with blocked protein-G agarose and washed extensively. Chromatin was eluted from the antibody by incubating for 10 min at room temperature with elution buffer (0.1 M NaHCO_3_ and 1% SDS). The crosslinking was reversed by adding NaCl to 0.2 M and incubating at 65 °C for at least 4 h. Samples were then digested with 40 ng of proteinase K, and the DNA was isolated via phenol/chloroform extraction followed by ethanol precipitation. DNA concentrations were calculated by measuring A260 on a NanoDrop ND-1000 spectrophotometer (NanoDrop, Wilmington, Delaware, United States). Sheared genomic DNA was generated by sonicating genomic DNA purified from CD1 mouse liver.

QPCR to determine enrichment of genomic loci was performed as for expression analysis, except that the reaction was performed on immunoprecipitated DNA. All occupancy calculations are the average of five samples in both the treated and fed control groups, with all QPCR reactions performed in triplicate. The fold-enrichment and *p*-value calculations were performed with the REST software package, with 2,000 randomizations. As our normalizing sequence, we used the loci encoding the 28S ribosomal RNA, a sequence of DNA present in multiple copies in the mouse genome. If no product was detected in a control sample in two of three triplicate wells after 45 cycles of PCR, 45 was used as the threshold cycle crossing point. Primer sequences are provided in Table S2.

### Location analysis.

For location analysis, a common reference design was utilized, where the common reference was sheared genomic DNA. The three immunoprecipitated treatment samples showing the largest enrichment for the known GR targets in the promoters/enhancers of the *Tat* and *Mt2* genes were used for location analysis and were compared to immunoprecipitations from five fed control samples. This resulted in a total of eight microarray hybridizations. Approximately one half of the material from an immunoprecipitation was amplified using an LM-PCR protocol modified from that developed by Oberley and colleagues [75]. The DNA was blunt-ended in a 50 μl reaction using 5 U of T4 DNA Polymerase (Promega, Madison, Wisconsin, United States), 1× T4 DNA polymerase buffer, and 400 μM dNTPs. The samples were incubated at 11 °C for 15 min, then purified using MinElute columns (Qiagen, Valencia, California, United States) per the manufacturer's protocol. The oligonucleotides OJW102 and OJW103 were annealed to produce a directional linker, which was blunt-ligated to the blunt-ended ChIP DNA in a 50 μl reaction containing 1 μl of high-concentration T4 DNA ligase (New England Biolabs, Beverly, Massachusetts, United States), 1× T4 Ligase buffer, and 0.9 μM annealed OJW102/OJW103. The reaction was incubated at 16 °C overnight and then purified using QiaQuick columns (Qiagen). The samples were PCR amplified in a 50 μl PCR reaction containing 5 U Taq DNA Polymerase (Promega), 1× Taq Polymerase buffer, 2 mM MgCl_2_, 400 μM dNTPs, and 1 μM OJW102 at the following cycling parameters: 1 cycle at 55 °C for 2 min and 72 °C for 5 min; then 15 cycles of 95 °C for 30 s, 55 °C for 30 s, and 72 °C for 1 min; then a final extension at 72 °C for 5 min. This first round of LM-PCR was purified using QiaQuick columns and then quantified using spectrophotometry. The second round of LM-PCR was performed with 100 ng of the first round product and identical PCR settings except that only ten amplification cycles were performed. This produced 3–4 μg of DNA.

Amplified material (1 μg) was labeled and hybridized against 1 μg of sheared genomic DNA. Samples were labeled using Ready-To-Go DNA labeling beads (Amersham, Little Chalfont, United Kingdom) per manufacturer's instructions. Briefly, water was added to the sample to a volume of 45 μl. The DNA was denatured at 95 °C for 3 min, then cooled on ice. Next, 5 μl of dCTP coupled to Cy3 or Cy5 dye (Amersham) was added, along with the Ready-To-Go labeling bead. This was gently mixed and incubated at 37 °C for 30 min. The Cy3 and Cy5 samples were combined and purified using MinElute columns (Qiagen). After purification, 500 ng of Cot1 Mouse DNA was added to each sample and denatured at 95 °C for 5 min. The samples were then cooled to 42 °C and an equal volume of 2× hybridization buffer (50% formamide, 10× SSC, and 0.2% SDS) was added, mixed, and applied to the Mouse PromoterChip BCBC-3.0 microarray slide.

Microarray slides were hybridized overnight, then washed and scanned with Agilent G2565BA Microarray Scanner. Images were analyzed with GenePix 5.0 software (Axon Instruments). Median background subtracted intensities were obtained for each spot and imported into the mathematical software package “R.” M and A values were calculated as log_2_(sample/control) and (log_2_ [sample] + log_2_ [control])/2, respectively. M values were Lowess-normalized with the SMA package developed by Speed and colleagues [71], and M values for the duplicate spots present on the array were averaged whenever both spots were present.

We used two criteria to determine whether the binding data indicated that a particular promoter region was enriched in the GR-immunoprecipitated material from the treatment samples. We first used SAM to determine the promoter regions differentially enriched between the treatment and control samples. SAM produced a list of differentially bound promoters, the majority of which were enriched in the treatment samples. Then we required that the dexamethasone-injected samples had to show enrichment compared to the sheared, unenriched genomic DNA by utilizing an average M value cutoff of M > 0.

### Statistical significance.

The probability of obtaining eight or more GR targets from the list of 12 known targets present on the promoter array was calculated using the hypergeometric distribution. The family of cyclin-dependent kinases and cytochrome P450 enzymes were not considered, because the source did not list individual family members.

### EASE functional category analysis.

The Expression Analysis Systematic Explorer (EASE) annotation tool provided by the National Center for Biotechnology Information (NCBI; http://apps1.niaid.nih.gov/david/) was utilized to determine GO Biological Function categories that are enriched in the set of 302 GR target genes compared to the entire set of genes on the promoter microarray. Also shown is a metric known as the “EASE score.” This is calculated as the upper bound of the distribution of jackknife Fisher exact probabilities. This score is a conservative adjustment of *p*-values generated by the Fisher exact test that penalizes the significance of categories supported by few genes and negligibly penalizes categories supported by many genes.

### Pathways analysis.

The network was generated using Ingenuity Pathways Analysis (Ingenuity Systems, http://www.ingenuity.com). The NCBI reference sequence (RefSeq) identifier for the 53 DEB genes, plus the GR itself, was utilized to seed the network generation algorithm. The algorithm produced only two networks with more than four genes, which were then merged into a single network.

### Sequence analysis.

The set of vertebrate transcription factor PWMs in TRANSFAC v7.3 and JASPAR was scored against the promoters in the DEB set and a background set of 1,120 other promoters randomly selected from those present on the Mouse PromoterChip BCBC-3.0 promoter microarray. The background set of promoters contained the same proportion of 1-kb tiles (tile 1) and 2-kb tiles (tile 2) as the DEB set. The results are the average of three runs with different background sets. The ability of each PWM, or combination of PWMs, to differentiate between the DEB and the background sets was calculated by measuring the area under the curve in a plot of sensitivity versus 1 − specificity (false positive rate) at different score thresholds. In the case of a combination of PWMs, the threshold scores of both PWMs, as well as the distance between the two, were varied to generate the curve. The optimal spacing and scoring were determined by choosing the parameter values that yielded the largest positive difference between the sensitivity and the false positive rate. The spacing reported is the distance between the outer ends of the binding sites. Values of *p* were calculated using the one-sided two-sample proportion test (“prop.text” function in the software package “R”) to determine the significance of the difference between the true positive and false positive rates. For the analysis of all combinations of GR and other matrices, we corrected for multiple testing by using a Bonferroni correction, setting the threshold for significance to *p* < 4 × 10^−5^ (0.05/1,145 combinations).

## Supporting Information

Table S1 Location Analysis Results Immunoprecipitations with antiserum against GR were performed on chromatin from livers of dexamethasone-treated mice and fed controls. The samples were amplified using LM-PCR and then hybridized to the Mu7K promoter microarray, using sheared genomic DNA as common control. Location analysis resulted in 318 promoters representing 302 genes that were differentially bound by the activated GR. We have listed the RefSeq identifier, a short description of the gene, and the increase in occupancy as measured by the microarray. Some genes are listed more than once, due to both tile 1 and tile 2 showing enrichment. This indicates either multiple GR binding sites or a single GR binding site near the overlap of the two tiles.
(119 KB DOC)

Table S2 Primer Sequences Primers were designed to amplify putative GREs within the promoter regions enriched in the location analysis. These were utilized in QPCR reactions as described in Materials and Methods.
(40 KB DOC)

Dataset S1 Complete Expression Data This table contains the annotated expression data for the five treatment (D6–D10) and control (F6–F10) samples. Hybridizations were carried out with a common reference design, where the common reference was a mixture of all samples. Values are expressed as Log (red/green), where the common reference sample was labeled in red and the test samples were labeled in green. Also included are fold-change calculations and a column indicating whether SAM software indicated that the spot was differentially expressed.
(10.5 MB XLS)

Dataset S2 Complete Location Analysis Data This table contains the annotated location analysis data for the three treatment (D8–D10) and control (F6–F10) samples used in our calculations. Hybridizations were carried out with a common reference design, where the common reference was sheared genomic DNA. Values are expressed as Log (red/green), where the common reference sample was labeled in red and the test samples were labeled in green.
(2.3 MB XLS)

## Abbreviations

AP-1: activating protein-1
ChIP: chromatin immunoprecipitation
DEB: differentially expressed, GR bound
EASE: Expression Analysis Systematic Explorer
GO: Gene Ontology Biological Function
GR: glucocorticoid receptor
GRE: glucocorticoid response element
GRU: glucocorticoid response unit
HSP: heat shock protein
kb: kilobase(s)
NCBI: National Center for Biotechnology Information
NF-κB: nuclear factor kappa B
OPG: osteoprotegerin
PWM: position-specific weight matrix
QPCR: quantitative real-time PCR
RefSeq: NCBI reference sequence
RT-QPCR: reverse-transcription quantitative real-time PCR
SACO: serial analysis of chromatin occupancy
