# Supplementary material for: Glucocorticoid Receptor-Dependent Gene Regulatory Networks
Source: PLoS Genet. 2005 Aug 5;1(2):e16. doi: 10.1371/journal.pgen.0010016 (PMC1186734; doi:10.1371/journal.pgen.0010016)
Supplement: Supplementary file 1 [file pgen.0010016.st001.doc]

SUPPLEMENTARY TABLE 1

| Refseq | Annotation | enrichment |
| --- | --- | --- |
| NM_011863 | Papss1 | 1.33 |
| NM_008256 | Hmgcs2 | 1.67 |
| NM_013798 | actin, gamma, cytoplasmic (Actg), | 1.42 |
| NM_018829 | adaptor-related protein complex 3, mu 1 subunit (Ap3m1), | 2.02 |
| NM_007405 | adenylate cyclase 6 (Adcy6), | 1.35 |
| NM_019822 | adhesion regulating molecule 1 (Adrm1), | 1.40 |
| NM_007408 | adipose differentiation related protein (Adfp), | 1.75 |
| NM_009627 | adrenomedullin (Adm), | 1.28 |
| NM_007409 | alcohol dehydrogenase 1 (class I) (Adh1), | 1.92 |
| NM_007437 | aldehyde dehydrogenase family 3, subfamily A2 (Aldh3a2), | 1.44 |
| NM_007437 | aldehyde dehydrogenase family 3, subfamily A2 (Aldh3a2), | 1.65 |
| NM_007423 | alpha fetoprotein (Afp), | 1.29 |
| NM_007585 | annexin A2 (Anxa2), | 1.38 |
| NM_007463 | aortic preferentially expressed gene 1 (Apeg1), | 1.43 |
| NM_013474 | apolipoprotein A-II (Apoa2), | 1.60 |
| NM_013474 | apolipoprotein A-II (Apoa2), | 1.57 |
| NM_138310 | apolipoprotein B48 receptor (Apob48r-pending), | 1.57 |
| NM_007469 | apolipoprotein C-I (Apoc1), | 1.59 |
| NM_007482 | arginase 1, liver (Arg1), | 1.47 |
| NM_011791 | ash2 (absent, small, or homeotic)-like (Drosophila) (Ash2l), | 1.80 |
| NM_009727 | ATPase, aminophospholipid transporter (APLT), (Atp8a1), | 2.09 |
| NM_015731 | ATPase, class II, type 9A (Atp9a), | 1.47 |
| NM_018794 | ATPase, H+ transporting (Atp6ip1), | 1.32 |
| NM_018731 | ATPase, H+/K+ transporting, alpha polypeptide (Atp4a), | 1.71 |
| NM_013454 | ATP-binding cassette, sub-family A (ABC1), member 1 (Abca1) | 1.86 |
| NM_013454 | ATP-binding cassette, sub-family A (ABC1), member 1 (Abca1) | 1.71 |
| NM_009735 | beta-2 microglobulin (B2m) | 1.92 |
| NM_009758 | bone morphogenetic protein receptor, type 1A (Bmpr1a) | 1.37 |
| NM_010238 | bromodomain containing 2 (Brd2) | 1.36 |
| NM_009922 | calponin 1 (Cnn1) | 1.57 |
| NM_007594 | calumenin (Calu) | 1.92 |
| NM_133828 | cAMP responsive element binding protein 1 (Creb1) | 1.43 |
| NM_009974 | casein kinase II, alpha 2, polypeptide (Csnk2a2) | 1.50 |
| NM_009804 | catalase (Cat) | 1.40 |
| NM_007744 | catechol-O-methyltransferase (Comt) | 1.70 |
| NM_016904 | CDC28 protein kinase 1 (Cks1) | 1.52 |
| NM_012000 | ceroid-lipofuscinosis, neuronal 8 (Cln8) | 1.71 |
| NM_009912 | chemokine (C-C motif) receptor 1 (Ccr1) | 1.36 |
| NM_013885 | chloride intracellular channel 4 (mitochondrial) (Clic4) | 2.34 |
| NM_009890 | cholesterol 25-hydroxylase (Ch25h) | 1.76 |
| NM_009881 | chromodomain protein, Y chromosome-like (Cdyl) | 1.37 |
| NM_007715 | circadian locomoter output cycles kaput (Clock) | 2.76 |
| NM_026444 | citrate synthase (Cs) | 1.72 |
| NM_007977 | coagulation factor VIII (F8) | 2.11 |
| NM_021489 | coagulation factor XII (Hageman factor) (F12) | 2.02 |
| NM_021538 | coatomer protein complex, subunit epsilon (Cope) | 2.48 |
| NM_023143 | complement component 1, r subcomponent (C1r) | 1.91 |
| NM_023143 | complement component 1, r subcomponent (C1r) | 1.37 |
| NM_022309 | core binding factor beta (Cbfb) | 1.53 |
| NM_022309 | core binding factor beta (Cbfb) | 1.36 |
| NM_018856 | cyclin L2 (Ccnl2) | 1.28 |
| NM_028850 | cysteine-rich hydrophobic domain 2 (Chic2) | 1.68 |
| NM_019779 | cytochrome P450, (Cyp11a1) | 1.59 |
| NM_010003 | cytochrome P450, (Cyp2c39) | 1.66 |
| NM_010006 | cytochrome P450, (Cyp2d9) | 2.07 |
| NM_022434 | cytochrome P450, (Cyp4f14) | 1.80 |
| NM_009895 | cytokine inducible SH2-containing protein (Cish) | 1.79 |
| NM_018776 | cytokine receptor-like factor 3 (Crlf3) | 1.44 |
| NM_016974 | D site albumin promoter binding protein (Dbp) | 1.30 |
| NM_015735 | damage specific DNA binding protein 1 (Ddb1) | 1.99 |
| NM_007840 | DEAD (Asp-Glu-Ala-Asp) box polypeptide 5 (Ddx5) | 1.43 |
| NM_010028 | DEAD/H (Asp-Glu-Ala-Asp/His) box polypeptide 3 (Ddx3) | 2.30 |
| NM_007828 | death-associated kinase 3 (Dapk3) | 1.25 |
| NM_007853 | degenerative spermatocyte homolog (Drosophila) (Degs) | 1.35 |
| NM_007830 | diazepam binding inhibitor (Dbi) | 1.49 |
| NM_007861 | dihydrolipoamide dehydrogenase (Dld) | 1.22 |
| NM_023047 | dihydropyrimidinase-like 5 (Dpysl5) | 1.24 |
| NM_010091 | dishevelled, dsh homolog 1 (Drosophila) (Dvl1) | 1.49 |
| NM_007859 | DNA fragmentation factor, beta subunit (Dffb) | 1.32 |
| NM_026187 | DNA segment, Chr 1, ERATO Doi 161, expressed (D1Ertd161e) | 3.14 |
| NM_007837 | DNA-damage inducible transcript 3 (Ddit3) | 1.46 |
| NM_026268 | dual specificity phosphatase 6 (Dusp6) | 1.52 |
| NM_026268 | dual specificity phosphatase 6 (Dusp6) | 1.35 |
| NM_007924 | eleven-nineteen lysine-rich leukemia gene (Ell) | 1.69 |
| NM_007932 | endoglin (Eng) | 1.40 |
| NM_010107 | ephrin A1 (Efna1) | 2.04 |
| NM_007909 | ephrin A2 (Efna2) | 1.36 |
| NM_010110 | ephrin B1 (Efnb1) | 1.31 |
| NM_010110 | ephrin B1 (Efnb1) | 1.77 |
| NM_010129 | epithelial membrane protein 3 (Emp3) | 1.36 |
| NM_015766 | Epstein-Barr virus induced gene 3 (Ebi3) | 1.37 |
| NM_019427 | erythrocyte protein band 4.1-like 4b (Epb4.1l4b) | 1.39 |
| NM_013515 | erythrocyte protein band 7.2 (Epb7.2) | 1.23 |
| NM_019480 | estrogen receptor-binding fragment-associated gene 9 (Ebag9) | 2.04 |
| NM_023060 | eukaryotic elongation factor, (Eefsec) | 1.32 |
| NM_026114 | eukaryotic translation initiation factor 2, subunit 1 alpha (Eif2s1) | 2.02 |
| NM_023314 | eukaryotic translation initiation factor 4E like 3 (Eif4el3) | 1.45 |
| NM_015769 | excision repair cross-complementing, (Ercc4) | 1.52 |
| NM_008033 | Farnesyltransferase, CAAX box, alpha (Fnta) | 1.48 |
| NM_007981 | fatty acid Coenzyme A ligase, long chain 2 (Facl2) | 1.31 |
| NM_010234 | FBJ osteosarcoma oncogene (Fos) | 1.39 |
| NM_015821 | F-box and leucine-rich repeat protein 8 (Fbxl8) | 1.59 |
| NM_021355 | fibromodulin (Fmod) | 1.22 |
| NM_023907 | forkhead box I1 (Foxi1) | 1.86 |
| NM_008057 | frizzled homolog 7 (Drosophila) (Fzd7) | 1.95 |
| NM_011356 | frizzled-related protein (Frzb) | 1.48 |
| NM_010176 | fumarylacetoacetate hydrolase (Fah) | 1.58 |
| NM_008092 | GATA binding protein 4 (Gata4) | 1.74 |
| NM_020567 | geminin (Gmnn) | 1.33 |
| NM_013847 | glycine C-acetyltransferase (Gcat) | 1.82 |
| NM_013847 | glycine C-acetyltransferase (Gcat) | 1.39 |
| NM_010321 | glycine N-methyltransferase (Gnmt) | 1.27 |
| NM_030678 | glycogen synthase 1, muscle (Gys1) | 1.86 |
| NM_008195 | glycogen synthase 3, brain (Gys3) | 1.43 |
| NM_013755 | glycogenin 1 (Gyg1) | 1.43 |
| NM_016697 | glypican 3 (Gpc3) | 1.60 |
| NM_008086 | growth arrest specific 1 (Gas1) | 1.41 |
| NM_019518 | GRP1 -associated scaffold protein (Grasp) | 1.96 |
| NM_008211 | H3 histone, family 3B (H3f3b) | 1.57 |
| NM_019479 | hairy and enhancer of split 6 (Drosophila) (Hes6) | 1.92 |
| NM_008213 | heart and neural crest derivatives expressed transcript 1 (Hand1) | 1.37 |
| NM_008302 | heat shock protein 1, beta (Hspcb) | 1.24 |
| NM_008225 | hematopoietic cell specific Lyn substrate 1 (Hcls1) | 1.61 |
| NM_010447 | heterogeneous nuclear ribonucleoprotein A1 (Hnrpa1) | 2.07 |
| NM_010439 | high mobility group box 1 (Hmgb1) | 1.40 |
| NM_023294 | highly expressed in cancer, (Hec-pending) | 1.64 |
| NM_008264 | Homeo box A13 (Hoxa13) | 1.26 |
| NM_010459 | Homeo box B4 (Hoxb4) | 1.59 |
| NM_008271 | homeo box C5 (Hoxc5) | 1.44 |
| NM_008272 | homeo box C9 (Hoxc9) | 1.59 |
| NM_010420 | homeo box gene expressed in ES cells (Hesx1) | 1.31 |
| NM_022331 | homocysteine-inducible, ER stress-inducible, (Herpud1) | 2.22 |
| NM_013547 | homogentisate 1, 2-dioxygenase (Hgd) | 2.31 |
| NM_010489 | hyaluronidase 2 (Hyal2) | 1.68 |
| NM_053262 | hydroxysteroid (17-beta) dehydrogenase 11 (Hsd17b11) | 1.40 |
| NM_019657 | hydroxysteroid (17-beta) dehydrogenase 12 (Hsd17b12) | 1.28 |
| NM_008292 | hydroxysteroid (17-beta) dehydrogenase 4 (Hsd17b4) | 1.34 |
| NM_008294 | hydroxysteroid dehydrogenase (Hsd3b4) | 1.63 |
| NM_010499 | immediate early response 2 (Ier2) | 1.50 |
| NM_023503 | inhibitor of growth family, member 1-like (Ing1l) | 1.34 |
| NM_027397 | insulin related protein 2 (islet 2) (Isl2) | 1.51 |
| NM_008341 | insulin-like growth factor binding protein 1 (Igfbp1) | 1.60 |
| NM_010577 | integrin alpha 5 (fibronectin receptor alpha) (Itga5) | 1.78 |
| NM_008407 | inter-alpha trypsin inhibitor, heavy chain 3 (Itih3) | 1.29 |
| NM_008394 | interferon dependent transcription factor 3 gamma (Isgf3g) | 1.30 |
| NM_010550 | interleukin 11 receptor, alpha chain 2 (Il11ra2) | 1.58 |
| NM_008368 | interleukin 2 receptor, beta chain (Il2rb) | 1.47 |
| NM_010492 | islet cell autoantigen 1 (Ica1) | 1.58 |
| NM_010497 | isocitrate dehydrogenase 1 (NADP+), soluble (Idh1) | 2.25 |
| NM_008323 | isocitrate dehydrogenase 3 (NAD+), gamma (Idh3g) | 1.62 |
| NM_008436 | K+ voltage-gated channel, subfamily S, 2 (Kcns2) | 2.05 |
| NM_010631 | kinesin family member C3 (Kifc3) | 1.58 |
| NM_023125 | kininogen (Kng) | 2.69 |
| NM_023184 | Kruppel-like factor 15 (Klf15) | 2.35 |
| NM_008453 | Kruppel-like factor 3 (basic) (Klf3) | 3.33 |
| NM_025808 | leucine-zipper-like transcriptional regulator, 1 (Lztr1) | 2.29 |
| NM_008280 | lipase, hepatic (Lipc) | 1.62 |
| NM_010719 | lipase, hormone sensitive (Lipe) | 1.88 |
| NM_133198 | liver glycogen phosphorylase (Pygl) | 1.29 |
| NM_016869 | low density lipoprotein receptor-related protein 4 (Lrp4) | 1.47 |
| NM_008529 | lymphocyte antigen 6 complex, locus E (Ly6e) | 1.33 |
| NM_010832 | male-specific lethal-3 homolog 1 (Drosophila) (Msl31) | 1.34 |
| NM_021462 | MAP kinase-interacting serine/threonine kinase 2 (Mknk2) | 1.29 |
| NM_010813 | max binding protein (Mnt) | 2.19 |
| NM_008621 | membrane protein, palmitoylated (Mpp1) | 1.70 |
| NM_008590 | mesoderm specific transcript (Mest) | 1.52 |
| NM_008638 | methenyltetrahydrofolate cyclohydrolase (Mthfd2) | 1.42 |
| NM_020577 | methyltransferase Cyt19 (Cyt19-pending) | 1.54 |
| NM_019758 | mitochondrial carrier homolog 2 (C. elegans) (Mtch2) | 1.47 |
| NM_025302 | mitochondrial ribosomal protein L2 (Mrpl2) | 1.48 |
| NM_011941 | mitogen activated protein kinase binding proten 1 (Mapkbp1) | 2.01 |
| NM_011844 | monoglyceride lipase (Mgll) | 1.44 |
| NM_020282 | NAD(P)H dehydrogenase, quinone 2 (Nqo2) | 1.39 |
| NM_023202 | NADH dehydrogenase (ubiquinone), (B14.5ª) (Ndufa7) | 1.40 |
| NM_026703 | NADH dehydrogenase (ubiquinone), (Ndufa8) | 2.09 |
| NM_008676 | neighbor of Brca1 gene 1 (Nbr1) | 2.61 |
| NM_008683 | neural precursor cell expressed, …, (Nedd8) | 1.56 |
| NM_008730 | neuronal pentraxin 1 (Nptx1) | 1.76 |
| NM_008667 | Ngfi-A binding protein 1 (Nab1) | 1.40 |
| NM_008697 | ninein (Nin) | 1.53 |
| NM_013865 | N-myc downstream regulated 3 (Ndr3) | 1.53 |
| NM_016813 | nuclear RNA export factor 1 homolog (S. cerevisiae) (Nxf1) | 1.23 |
| NM_019801 | open reading frame 18 (ORF18) | 1.70 |
| NM_016978 | ornithine aminotransferase (Oat) | 1.51 |
| NM_013614 | ornithine decarboxylase, structural (Odc) | 1.53 |
| NM_011134 | paraoxonase 1 (Pon1) | 1.33 |
| NM_016861 | PDZ and LIM domain 1 (elfin) (Pdlim1) | 1.41 |
| NM_009343 | PHD finger protein 1 (Phf1) | 1.34 |
| NM_011044 | phosphoenolpyruvate carboxykinase 1, cytosolic (Pck1) | 2.50 |
| NM_011044 | phosphoenolpyruvate carboxykinase 1, cytosolic (Pck1) | 2.30 |
| NM_025801 | phosphogluconate dehydrogenase (Pgd) | 1.60 |
| NM_020272 | phosphoinositide-3-kinase, (Pik3cg) | 1.51 |
| NM_054066 | phospholipase C, zeta 1 (Plcz1) | 1.35 |
| NM_016881 | phosphomannomutase 2 (Pmm2) | 1.23 |
| NM_008877 | plasminogen (Plg) | 1.74 |
| NM_019549 | pleckstrin (Plek) | 1.34 |
| NM_138674 | polycystic kidney and hepatic disease 1-like 1 (Pkhd1l1) | 1.35 |
| NM_011132 | polymerase (DNA directed), epsilon (Pole) | 1.33 |
| NM_008956 | polypyrimidine tract binding protein 1 (Ptbp1) | 1.60 |
| NM_134110 | potassium voltage-gated channel, (Kcne2) | 1.34 |
| NM_010597 | potassium voltage-gated channel, (Kcnab1) | 1.37 |
| NM_011070 | prefoldin 2 (Pfdn2) | 1.97 |
| NM_153393 | procollagen, type XXIII, alpha 1 (Col23a1) | 1.34 |
| NM_019746 | programmed cell death 5 (Pdcd5) | 1.41 |
| NM_008831 | prohibitin (Phb) | 1.31 |
| NM_016883 | proteasome (prosome, macropain) 26S subunit, (Psmd10) | 1.70 |
| NM_008951 | proteasome (prosome, macropain) 26S subunit, (Psmd4) | 1.33 |
| NM_010817 | proteasome (prosome, macropain) 26S subunit, (Psmd7) | 1.72 |
| NM_011968 | proteasome (prosome, macropain) subunit, (Psma6) | 1.51 |
| NM_011862 | PKC and casein kinase substrate in neurons 2 (Pacsin2) | 1.87 |
| NM_153745 | protein kinase, AMP-activated, (Prkag3) | 1.64 |
| NM_008014 | protein phosphatase 1G (formerly 2C), (Ppm1g) | 1.27 |
| NM_011154 | protein phosphatase 2 (formerly 2A), (Ppp2r3a) | 1.50 |
| NM_009358 | protein phosphatase 2, regulatory subunit B (B56), (Ppp2r5d) | 1.21 |
| NM_008974 | protein tyrosine phosphatase 4a2 (Ptp4a2) | 1.25 |
| NM_011203 | protein tyrosine phosphatase, non-receptor type 12 (Ptpn12) | 1.68 |
| NM_009419 | protein-tyrosine sulfotransferase 2 (Tpst2) | 1.34 |
| NM_008771 | purinergic receptor P2X, ligand-gated ion channel, 1 (P2rx1) | 1.42 |
| NM_019698 | pyrroline-5-carboxylate synthetase (Pycs) | 1.53 |
| NM_133667 | pyruvate dehydrogenase kinase, isoenzyme 2 (Pdk2) | 1.54 |
| NM_011231 | RAB geranylgeranyl transferase, b subunit (Rabggtb) | 1.57 |
| NM_017382 | RAB11a, member RAS oncogene family (Rab11a) | 1.34 |
| NM_012025 | Rac GTPase-activating protein 1 (Racgap1) | 1.27 |
| NM_023275 | ras homolog gene family, member J (Arhj) | 1.50 |
| NM_025846 | related RAS viral (r-ras) oncogene homolog 2 (Rras2) | 1.64 |
| NM_020022 | replication factor C (activator 1) 2 (Rfc2) | 1.89 |
| NM_020509 | resistin like alpha (Retnla) | 1.39 |
| NM_009050 | ret proto-oncogene (Ret) | 1.24 |
| NM_019647 | ribosomal protein L21 (Rpl21) | 1.72 |
| NM_019647 | ribosomal protein L21 (Rpl21) | 1.47 |
| NM_011296 | ribosomal protein S18 (Rps18) | 1.29 |
| NM_008503 | ribosomal protein S2 (Rps2) | 1.46 |
| NM_016959 | ribosomal protein S3a (Rps3a) | 1.22 |
| NM_009098 | ribosomal protein S8 (Rps8) | 1.28 |
| NM_025356 | RIKEN cDNA 1100001F19 gene (1100001F19Rik) | 1.97 |
| NM_029565 | RIKEN cDNA 1110001M20 gene (1110001M20Rik) | 1.55 |
| NM_025368 | RIKEN cDNA 1110007C05 gene (1110007C05Rik) | 1.83 |
| NM_026124 | RIKEN cDNA 1110008F13 gene (1110008F13Rik) | 1.69 |
| NM_023168 | RIKEN cDNA 1110025J15 gene (1110025J15Rik) | 1.92 |
| NM_025836 | RIKEN cDNA 1300012C15 gene (1300012C15Rik) | 1.65 |
| NM_025919 | RIKEN cDNA 2010203J19 gene (2010203J19Rik) | 1.21 |
| NM_026438 | RIKEN cDNA 2010317E03 gene (2010317E03Rik) | 1.25 |
| NM_023712 | RIKEN cDNA 2210013K02 gene (2210013K02Rik) | 1.47 |
| NM_025963 | RIKEN cDNA 2210402A09 gene (2210402A09Rik) | 2.20 |
| NM_025519 | RIKEN cDNA 2310010I16 gene (2310010I16Rik) | 1.42 |
| NM_026507 | RIKEN cDNA 2310031L18 gene (2310031L18Rik) | 1.98 |
| NM_026509 | RIKEN cDNA 2310039E09 gene (2310039E09Rik) | 1.59 |
| NM_026403 | RIKEN cDNA 2610027L16 gene (2610027L16Rik) | 1.76 |
| NM_026522 | RIKEN cDNA 3110023E09 gene (3110023E09Rik) | 1.28 |
| NM_024473 | RIKEN cDNA 8030460C05 gene (8030460C05Rik) | 1.68 |
| NM_016698 | ring finger protein 10 (Rnf10) | 1.19 |
| NM_013876 | ring finger protein 11 (Rnf11) | 1.53 |
| NM_019484 | RNA and export factor binding protein 2 (Refbp2) | 1.43 |
| NM_011251 | RNA binding motif protein 6 (Rbm6) | 1.76 |
| NM_020296 | RNA binding motif, (Rbms1) | 3.38 |
| NM_018872 | RW1 protein (Rw1-pending) | 1.85 |
| NM_009113 | S100 calcium binding protein A13 (S100a13) | 1.37 |
| NM_016661 | S-adenosylhomocysteine hydrolase (Ahcy) | 1.99 |
| NM_015772 | sal-like 2 (Drosophila) (Sall2) | 1.49 |
| NM_011328 | secretin (Sct) | 1.40 |
| NM_011370 | selective hybridizing clone (Shyc) | 2.25 |
| NM_019414 | selenium binding protein 2 (Selenbp2) | 1.62 |
| NM_009155 | selenoprotein P, plasma, 1 (Sepp1) | 1.21 |
| NM_013759 | selenoprotein R (Sepr) | 1.30 |
| NM_018884 | semaF cytoplasmic domain associated protein 3 (Semcap3-pending) | 3.93 |
| NM_009243 | serine (or cysteine) proteinase inhibitor, (Serpina1a) | 1.69 |
| NM_009243 | serine (or cysteine) proteinase inhibitor, (Serpina1a) | 1.67 |
| NM_008878 | serine (or cysteine) proteinase inhibitor, (Serpinf2) | 2.56 |
| NM_008878 | serine (or cysteine) proteinase inhibitor, (Serpinf2) | 1.57 |
| NM_009250 | serine (or cysteine) proteinase inhibitor, (Serpini1) | 1.61 |
| NM_019464 | SH3-domain GRB2-like B1 (endophilin) (Sh3glb1) | 1.75 |
| NM_021448 | STAT interacting protein 1 (Statip1) | 2.13 |
| NM_026506 | small nuclear ribonucleoprotein polypeptide G (Snrpg) | 1.59 |
| NM_018732 | sodium channel, voltage-gated, (Scn3a) | 1.46 |
| NM_018861 | solute carrier family 1 (Slc1a4) | 1.88 |
| NM_009202 | solute carrier family 22 (Slc22a1) | 1.50 |
| NM_023214 | solute carrier family 30 (zinc transporter), (Slc30a7) | 2.42 |
| NM_025286 | solute carrier family 31, member 2 (Slc31a2) | 2.09 |
| NM_009231 | Son of sevenless homolog 1 (Drosophila) (Sos1) | 1.37 |
| NM_025287 | speckle-type POZ protein (Spop) | 1.64 |
| NM_007380 | spectrin SH3 domain binding protein 1 (Ssh3bp1) | 1.46 |
| NM_016882 | squamous cell carcinoma antigen recognized by T-cells 1 (Sart1) | 1.18 |
| NM_011368 | src homology 2 domain-containing (Shc1) | 2.42 |
| NM_011441 | SRY-box containing gene 17 (Sox17) | 1.46 |
| NM_025291 | steroid receptor RNA activator 1 (Sra1) | 1.43 |
| NM_019719 | STIP1 homology and U-Box containing protein 1 (Stub1) | 1.83 |
| NM_023231 | stomatin (Epb7.2)-like 2 (Stoml2) | 1.19 |
| NM_015752 | suppressor of fused homolog (Drosophila) (Sufu) | 1.30 |
| NM_009296 | suppressor of Ty 4 homolog (S. cerevisiae) (Supt4h) | 1.32 |
| NM_011602 | talin (Tln) | 1.51 |
| NM_009318 | TAP binding protein (Tapbp) | 1.65 |
| NM_011603 | TATA box binding protein-like 1 (Tbpl1) | 2.17 |
| NM_011603 | TATA box binding protein-like 1 (Tbpl1) | 1.51 |
| NM_011607 | tenascin C (Tnc) | 1.68 |
| NM_011660 | thioredoxin 1 (Txn1) | 1.51 |
| NM_011593 | tissue inhibitor of metalloproteinase 1 (Timp1) | 1.54 |
| NM_009410 | topoisomerase (DNA) III alpha (Top3a) | 2.07 |
| NM_009410 | topoisomerase (DNA) III alpha (Top3a) | 1.47 |
| NM_009328 | transcription factor 15 (Tcf15) | 1.37 |
| NM_011545 | transcription factor 21 (Tcf21) | 1.45 |
| NM_031182 | transcription factor AP-4 (Tfap4) | 1.28 |
| NM_020507 | transducer of ERBB2, 2 (Tob2) | 3.13 |
| NM_015799 | transferrin receptor 2 (Trfr2) | 1.34 |
| NM_011640 | transformation related protein 53 (Trp53) | 2.29 |
| NM_009367 | transforming growth factor, beta 2 (Tgfb2) | 1.67 |
| NM_009367 | transforming growth factor, beta 2 (Tgfb2) | 1.37 |
| NM_019828 | transient receptor potential cation channel, (Trpc4ap) | 2.17 |
| NM_012032 | tumor differentially expressed 1 (Tde1) | 1.21 |
| NM_013693 | tumor necrosis factor (Tnf) | 1.34 |
| NM_008764 | tumor necrosis factor receptor superfamily, (Tnfrsf11b) | 1.22 |
| NM_011609 | tumor necrosis factor receptor superfamily, (Tnfrsf1a) | 1.30 |
| NM_009536 | tyrosine 3-monooxygenase/tryptophan 5-monooxygenase activation protein, epsilon polypeptide (Ywhae) | 1.52 |
| NM_011738 | tyrosine 3-monooxygenase/tryptophan 5-monooxygenase activation protein, eta polypeptide (Ywhah) | 1.86 |
| NM_011740 | tyrosine 3-monooxygenase/tryptophan 5-monooxygenase activation protein, zeta polypeptide (Ywhaz) | 1.31 |
| NM_011432 | U1 small nuclear ribonucleoprotein 1C (Snrp1c) | 1.83 |
| NM_019729 | ubiquitin specific protease 8 (Usp8) | 1.59 |
| NM_019729 | ubiquitin specific protease 8 (Usp8) | 1.27 |
| NM_017377 | UDP-Gal:betaGlcNAc beta 1,4- galactosyltransferase (B4galt2) | 1.28 |
| NM_011673 | UDP-glucose ceramide glucosyltransferase (Ugcg) | 1.26 |
| NM_011551 | upstream binding transcription factor, RNA pol I (Ubtf) | 1.52 |
| NM_009474 | urate oxidase (Uox) | 1.73 |
| NM_016872 | vesicle-associated membrane protein 5 (Vamp5) | 1.45 |
| NM_011701 | vimentin (Vim) | 1.41 |
| NM_009524 | wingless-related MMTV integration site 5A (Wnt5a) | 1.49 |
| NM_020570 | X-ray repair complementing defective repair (Xrcc2) | 1.74 |
| NM_021394 | Z-DNA binding protein 1 (Zbp1) | 1.48 |
| NM_018759 | zinc finger protein 326 (Zfp326) | 1.74 |
| NM_011777 | zyxin (Zyx) | 1.60 |

Location Analysis Results. Immunoprecipitations with α-GR antiserum were performed on chromatin from dexamethasone-treated livers and fed controls. The samples were amplified using LM-PCR and then hybridized to the Mu7K Promoter microarray, using sheared genomic DNA as common control. Location Analysis resulted in 318 promoters representing 302 genes which were differentially bound by the activated glucocorticoid receptor. We have listed the Refseq identifier, a short description of the gene, and the increase in occupancy as measured by the microarray. Some genes are listed more than once, due to both Tile 1 and Tile 2 showing enrichment. This indicates either multiple GR binding sites or a single GR binding site near the overlap of the two tiles.
