# Supplementary material for: Glucocorticoid Receptor-Dependent Gene Regulatory Networks
Source: PLoS Genet. 2005 Aug 5;1(2):e16. doi: 10.1371/journal.pgen.0010016 (PMC1186734; doi:10.1371/journal.pgen.0010016)
Supplement: Supplementary file 2 [file pgen.0010016.st002.doc]

| Primer Sequences for ChIP on Genomic DNA | |  |  |
| --- | --- | --- | --- |
| Primer Set Name | Description | Forward Primer | Reverse Primer |
| 28S | Loading Control | ctgggtataggggcgaaagac | ggccccaagacctctaatcat |
| TAT | Near GRE in Tat promoter | cgcaaacaacaggaagcctaa | catgacacccaaaagcctctc |
| MT2 | Near GRE in Mt2 promoter | tgttacaacccacccacagc | tcatctgcagagccaggaaa |
| Pck | Near GRE in Pck promoter | tgcagccagcaacatatgaa | tgatgcaaactgcaggctct |
| apoa2 | Promoter of NM_013474 | gggcctagcgaatgtacctct | tcctgctcccaacctcagtag |
| Comt | Promoter of NM_007744 | acactcgggatctgtgtggat | atgggtcattgtgtccaggag |
| B2m | Promoter of NM_009735 | cccgattaagttctccatcca | ccctcctgtcctaaaggcaac |
| Klf3 | Promoter of NM_008453 | agaccagacatgccgttttgt | gggactggatcaaacatgagc |
| Klf15 | Promoter of NM_023184 | ctgagcccctcttggatacct | gtctgggacactcaccgactt |
| Spop | Promoter of NM_025287 | tgcctttcccagaaagaacag | aggtgataccgcgtcttgaaa |
| Serpina1 | Promoter of NM_009243 | caaagccaacccacttgtctc | ttaggcatgtgtgagcattgg |
| Ier2 | Promoter of NM_010499 | gcgtgtgagagcatgagagaa | ggtcgttcgagtctgtttggt |
| Ugcg | Promoter of NM_011673 | agcatggttctttcccactga | cctggtctacagagttccaggac |
| Opg | Promoter of NM_008764 | gccacttacacagggcttgaa | gaggaaggaaaagctgcatga |
| cat | Promoter of NM_009804 | tgatcctctggtttccacctg | gaggctgtggctcagaaggt |
| Thio | Promoter of NM_011660 | gtagggtggctgagacacagttt | gaactcaccaagcctgcattt |
| adrp | Promoter of NM_007408 | ttctggtgacaggggatctatgt | cctggaaaaagatacaggtgcag |
| Tyrs | Promoter of NM_009536 | atcaaaacagcttgcccagatag | gaacggacaggaattgttttgtc |
